# Control Law Design for Propofol Infusion to Regulate Depth of Hypnosis: A Nonlinear Control Strategy

**DOI:** 10.1155/2016/1810303

**Published:** 2016-04-27

**Authors:** Ali Khaqan, Muhammad Bilal, Muhammad Ilyas, Bilal Ijaz, Raja Ali Riaz

**Affiliations:** Department of Electrical Engineering, COMSATS Institute of Information Technology, Chak Shahzad, Park Road, Islamabad 44000, Pakistan

## Abstract

Maintaining the depth of hypnosis (DOH) during surgery is one of the major objectives of anesthesia infusion system. Continuous administration of Propofol infusion during surgical procedures is essential but increases the undue load of an anesthetist in operating room working in a multitasking setup. Manual and target controlled infusion (TCI) systems are not good at handling instabilities like blood pressure changes and heart rate variability arising due to interpatient variability. Patient safety, large interindividual variability, and less postoperative effects are the main factors to motivate automation in anesthesia. The idea of automated system for Propofol infusion excites the control engineers to come up with a more sophisticated and safe system that handles optimum delivery of drug during surgery and avoids postoperative effects. In contrast to most of the investigations with linear control strategies, the originality of this research work lies in employing a nonlinear control technique, backstepping, to track the desired hypnosis level of patients during surgery. This effort is envisioned to unleash the true capabilities of this nonlinear control technique for anesthesia systems used today in biomedical field. The working of the designed controller is studied on the real dataset of five patients undergoing surgery. The controller tracks the desired hypnosis level within the acceptable range for surgery.

## 1. Overview

The hypnosis profile of the drug is considered in three distinct time-based segments in order: induction, maintenance, and emergence. These steps are liable for transferring the patient conscious level to a value appropriate for surgery, maintaining the optimum amount of drug in the body for smooth conduction of surgery after which the drug infusion is terminated to obtain emergence phase. Emergence phase is responsible for bringing the patient to a fully awake state. The finding of diverse inhalational gases in the 19th century was the turning point in the area of medicine [1]. Trivial measures to bring the patient in unconscious state like application of cold, compression of nerve, or reduction in cerebral perfusion were used throughout surgical activities [2]. The excess as well as insufficient quantity of drug in patient indicates a disturbing situation like inadequate analgesia or awareness. Fast induction of drug may grow the discomfort interrelated with the Propofol infusion in conscious patients. With this problem under consideration, administration of drug is very substantial in modifying the drug amount in the patient body. The basic aim of anesthesia is to deliver painless feeling when the patient is in unconscious condition during surgery. The viewpoint of clinical surgery has been completely altered by modern medicine through the practice of scientific and technological evolutions in the biomedical field. This incredible breakthrough has been reached only through the research results gained in modern anesthesia. The utilization of intravenous anesthetic drugs has been increased in the last decade, as these agents can promote quick loss of wakefulness by directly targeting the vascular system and reach the central nervous system rapidly. Resulting from exposure to different anesthetic agents, intravenous anesthetic drugs have lower incidence of adverse effects. The most used hypnotic drug for general anesthesia in biomedical domain is Propofol because of its number of advantages [3]. The dosage pattern is then set by hit and trial to improve the amount of anesthesia in patient.

The practice of controlling intravenous drug delivery has been developed from simple manual delivery and computer-assisted automated target controlled infusion (TCI) to more refined Closed-Loop Anesthesia (CLAN) systems [4]. TCI relies on population-based pharmacokinetics (PK) and pharmacodynamics (PD) models for calculating a proper infusion profile to achieve the reference drug concentration set by the anesthesiologist [5]. Depending on the past and present infusion profiles, these model representations can forecast the time evolution of plasma concentration [6]. This estimation is then used to track the reference concentration, thus formulating an open-loop control paradigm. TCI systems suffer from drawbacks of sensitivity to model nonlinearities and disturbances [7].

Drug doses are reliant on patient demographics, qualitatively measured signs (e.g., presence of certain reflexes and movement) and quantitatively measured signals (e.g., oxygen saturation, blood pressure, and heart rate). Anesthesia skill and expertise play momentous role because of the ambiguity level related to this process. Typically, hypnotic drug delivery rates in intravenous anesthesia are physically observed by anesthetists.

**Figure 1:**
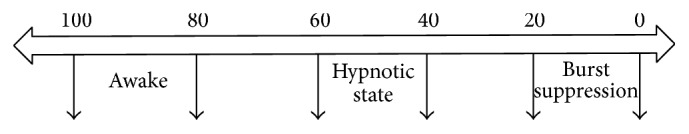
BIS scaling band to indicate DOH level.

Commercially available devices, like Bispectral Index (BIS), are extensively used to diagnose the depth of hypnosis for patients. The BIS is mapped to the DOH value of a patient based on the scaling band shown in Figure 1. The value of 100–80 corresponds to a fully awake state while band of 60–40 shows moderate hypnosis level [8]. The moderate level defines the surgical procedure band in which general surgery is completed by clinical professionals. Level beyond deep hypnotic state (40–20) is quite dangerous [9].

The way in which these anesthesia systems have been realized is based on linear control approaches [10]. In [11], employing PID, control performance has been observed with 10 patients experiencing knee surgery. The suggested control system was able to deliver suitable amount of anesthesia in 9 patients, while 3 patients showed oscillatory response in their BIS values. Some other noticeable studies showing control of anesthesia using PID include [12, 13]. Comparing orthodox PID with Linear Model Predictive Control (LMPC), it is investigated in [14] that the latter technique performs better in the sense of robustness to intra- and interpatient dynamics and handling unpredictable disturbances. Recent research efforts [15–18] aim to target linear approaches by accurately tuning the controllers to attain appropriate robustness margins for identifiable uncertainties. Such approximation attains good control performance only if the difference between the predicted and actual closed-loop systems is small for the designed controller [19]. The traditional PID controller cannot handle disturbances like blood pressure changes, neural muscular blockade, and heart rate variability [10] and may result in oscillatory performance during clinical trials.

Large interpatient variability and the output disturbances are the two main challenging features which may also be stimulated by the surgical equipment in operating room to achieve desired results. Some other limitations can also be enforced by the evidence that certain anesthetic drugs have adverse side effects. This suggests that drug infusion and maintenance must be restrictively examined throughout the entire surgical activity.

## 2. Pharmacokinetics-Pharmacodynamics Modeling

**Figure 2:**
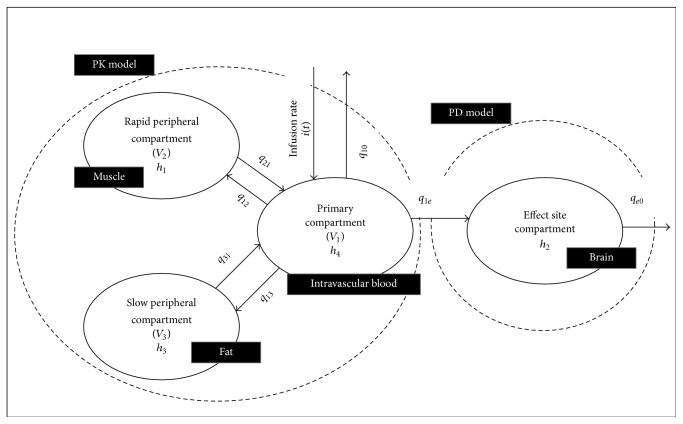
Integrated PKPD model.

The clinical behavior of the hypnotic drug (Propofol) is categorized in its pharmacokinetics (PK) and pharmacodynamics (PD) parameters. The PK parameters are intended to analyse the consequences of the drug in the body over a certain period of time including its distribution, metabolism, and clearance [20]. The concentration of drug in the blood and the corresponding impact initiated at the effect site are depicted by the use of PD parameter [21]. Due to fast redistribution and metabolism nature, the intravenously administered anesthetic drug like Propofol is used commonly during surgical activities. The drug concentrations and the drug effect data are measured simultaneously from the parameters of the PKPD models. In medical research, human body is distributed in different parts depending on the flow of blood [11]. This compartmental modelling describes the basic approach demonstrating the procedure of absorption, distribution, and elimination of the drug from the patient's body [13] and relating plasma-drug values to PD parameters. In this work, four-dimensional integrated PKPD model is used because of its adequate accuracy and computational efficacy [16]. The infusion and elimination of the drug between these compartments take place through the use of rate constants (*q*_12_, *q*_21_, *q*_13_, *q*_31_) as depicted in Figure 2 [17]. The arrangement of this compartmental modelling consists of three compartments with volumes *V*_1_, *V*_2_, and *V*_3_, respectively. The primary compartment represents the intravascular blood, rapid peripheral compartment shows the muscles, and the slow peripheral compartment denotes the fats in the body. The complexity of PKPD model can be enhanced by increasing the number of compartments showing the more detailed infusion profile of drug in patient's body.

At the effect site, the drug concentration is measured through the cortical activity in the brain determined through the processed EEG signal. The patient's brain activity is examined through EEG as the anesthetic drug affects the brain [13]. The pertinent material extracted from the EEG signal is then mapped to depth of hypnosis (DOH) to check the patient's state suitable for surgery. The infusion as well as clearance of the Propofol is done through the primary compartment in an exponential fashion [18].

In the last decade, the dynamic control of nonlinear systems has appeared as an exciting research field which constantly motivates the ideas of control engineers. This research is envisioned at unfolding the true capabilities of a nonlinear control scheme based on backstepping design to manage Propofol anesthesia infusion rate. This design procedure proves to be a powerful and effective tool which can be applied to a wide class of nonlinear systems operating in uncertain environment. It is a systematic Lyapunov method to design control algorithms which stabilize nonlinear systems [22].

This paper is arranged as follows.  Section 2 derives patient model.  Section 3 explains the design details of controller using backstepping, while Section 4 presents results and the summarizing discussion based on clinical parameters of actual patients. Finally, Section 5 draws the conclusion.

### 2.1. Patient Modelling via PKPD Approach

This part of the section shows differential equation model of how Propofol leads to hypnosis. State equations of the PK model corresponding to the three compartments of the integrated PKPD model can be written as using a three-compartment Schneider model as described in [8]:

(1) h˙4t=−q10h4t−q12h4t−q13h4t+q21h1t+q31h3t+it,

(2) h˙1t=q12h4t−q21h1t,

(3) h˙3t=q13h4t−q31h3t.

Taking Laplace transform of the above equations,

(4) sH4s=−q10+q12+q13H4s+q21H1s+q31H3s+Is,sH1s=q12H4s−q21H1s,sH3s=q13H4s−q31H3s.

From the above equations, the input-output relationship is given as

(5) $$\begin{array}{c} \frac{H_{4}\left(s\right)}{W\left(s\right)}=\frac{\left(s^{2}+s\left(q_{21}+q_{31}\right)+q_{21}q_{31}\right)}{\left(s^{3}+s^{2}\left(q_{10}+q_{12}+q_{21}+q_{13}+q_{31}\right)+s\left(q_{10}q_{21}+q_{10}q_{31}+q_{12}q_{31}+q_{13}q_{21}+q_{31}q_{21}\right)+q_{10}q_{21}q_{31}\right)}. \end{array}$$

PK model is generally written as

(6) $$\begin{array}{c} \frac{H_{4}\left(s\right)}{W\left(s\right)}=\frac{n_{2}s^{2}+n_{1}s+n_{0}}{d_{3}s^{3}+d_{2}s^{2}+d_{1}s+d_{0}}, \end{array}$$

where *n*_2_ = 1, *n*_1_ = *q*_21_ + *q*_31_, *n*_0_ = *q*_21_ *q*_31_, *d*_3_ = 1, *d*_2_ = (*q*_10_ + *q*_12_ + *q*_21_ + *q*_13_ + *q*_31_), *d*_1_ = (*q*_10_ *q*_21_ + *q*_10_ *q*_31_ + *q*_12_ *q*_31_ + *q*_13_ *q*_21_ + *q*_31_ *q*_21_), and *d*_0_ = *q*_10_ *q*_21_ *q*_31_.

State equation corresponding to PD model relates to concentration of the drug in plasma to the effect site concentration and can be modeled as

(7) $$\begin{array}{c} {\dot{h}}_{2}\left(\;t\;\right)=q_{1e}h_{4}\left(\;t\;\right)-q_{e0}h_{2}\left(\;t\;\right). \end{array}$$

Using Laplace transform on the above equation,

(8) $$\begin{array}{c} sH_{2}\left(\;s\;\right)=q_{1e}H_{4}\left(\;s\;\right)-q_{e0}H_{2}\left(\;s\;\right). \end{array}$$

Taking *q*_1*e*_ and *q*_*e*0_ as equals because of negligible volume at the effect site compartment, the overall PD model can be written as

(9) $$\begin{array}{c} \frac{H_{2}\left(s\right)}{H_{4}\left(s\right)}=\frac{q_{e0}}{\left(s+q_{e0}\right)}. \end{array}$$

From the cascaded behavior of PK and PD models, the complete patient model can finally be shown as

(10) $$\begin{array}{c} M_{p}\left(\;s\;\right)=\frac{q_{e0}*\left(n_{2}s^{2}+n_{1}s+n_{0}\right)}{\left(d_{3}s^{3}+d_{2}s^{2}+d_{1}s+d_{0}\right)\left(s+q_{e0}\right)}. \end{array}$$

The Bispectral Index is linked with effect site concentration *C*_*e*_(*t*) through nonlinear sigmoid model [16]:

(11) $$\begin{array}{c} \text{BIS}\left(\;t\;\right)={CE}_{0}-{CE}_{max}*\frac{{C_{e}\left(t\right)}^{\gamma }}{\left({C_{e}\left(t\right)}^{\gamma }+C_{50}^{\gamma }\right)}, \end{array}$$

where CE_0_ corresponds to the clinical effect without drug and CE_max_ is the maximum effect achieved [23] after drug infusion. The nomenclature used in deriving the patient model is shown in Nomenclature.

## 3. Control Law Design

Excessive or insufficient amount of drug infusion may lead to potentially deleterious effects on patient's health. The goal to design automated anesthesia system is patient's life safety. The prime function of the designed system is to administer the hypnosis level and analgesia of patient undergoing surgery by automating the initial amount of drug and the subsequent sustained infusion rate of drug.

### 3.1. Proposed Design

This segment presents the design of control law using a nonlinear control technique for optimum delivery of Propofol anesthesia during surgical procedures. The prime objective of this arrangement is to minimize the steady state error while maintaining the patient's DOH level in the required band suitable for surgery, as shown in Figure 1. The complete architecture of the designed system mainly constitutes a nonlinear controller cascaded with the patient PKPD model followed by a nonlinear sigmoid model.

Backstepping is a recursive design procedure used for designing stabilizing control for the class of nonlinear dynamical systems. This nonlinear control strategy consists of dividing a whole design problem into sequence of small problems of lower order [22]. This method ensures that the output tracks the desired reference signal during surgery to avoid complexities. One of the important features of this technique is its ability to tackle nonlinearities in a very special way; that is, instead of cancelling the useful nonlinearities present in the system, retaining them will give more benefits and may require less control effort [24]. The four states of our patient model are

(12) h˙1=a1h4−a2h1,

(13) h˙2=d1h2+d2h4,

(14) h˙3=b1h4−b2h3,

(15) h˙4=−c1h4+a2h1+b2h3+it.

As *a*_1_ = *q*_12_, *a*_2_ = *q*_21_, *b*_1_ = *q*_13_, *b*_2_ = *q*_31_, and *c*_1_ = (*q*_10_ + *q*_12_ + *q*_13_), *d*_1_ = *q*_*e*0_ = −0.456 and *d*_2_ = *q*_*e*0_ = 0.1068 are calculated [25] as shown at the end of this section. Defining the error variables for the four states with *h*_*d*_ as desired trajectory to be tracked, *α*_1_, *α*_2_, and *α*_3_ are the first, second, and third stabilizing functions:

(16) x1=h1−hd,

(17) x2=h2−α1,

(18) x3=h3−α2,

(19) x4=h4−α3.

**Step 1 .**

Assume the new variable *x*_1_ that shows the error between the actual output and desired output (BIS value) which can be represented by (16).

Taking time derivative of (16),

(20) $$\begin{array}{c} {\dot{x}}_{1}={\dot{h}}_{1}-{\dot{h}}_{d}. \end{array}$$

Putting (12) related to rapid peripheral compartment, we obtain

(21) $$\begin{array}{c} {\dot{x}}_{1}=-a_{2}h_{1}+a_{1}h_{4}-{\dot{h}}_{d}. \end{array}$$

Now using (16) and (19) in the above equation,

(22) $$\begin{array}{c} {\dot{x}}_{1}=-a_{2}\left(\;x_{1}+h_{d}\;\right)+a_{1}\left(\;x_{4}+\alpha_{3}\;\right)-{\dot{h}}_{d}. \end{array}$$

After simplification,

(23) $$\begin{array}{c} {\dot{x}}_{1}=-a_{2}x_{1}-a_{2}h_{d}+a_{1}x_{4}+a_{1}\alpha_{3}-{\dot{h}}_{d}. \end{array}$$

Assume the Lyapunov function as

(24) $$\begin{array}{c} f_{1}=0.5x_{1}^{2}. \end{array}$$

Derivative of the above equation is

(25) $$\begin{array}{c} {\dot{f}}_{1}=x_{1}{\dot{x}}_{1}. \end{array}$$

Solving for ${\dot{f}}_{1}$, substituting (23) in (25),

(26) $$\begin{array}{c} {\dot{f}}_{1}=-a_{2}x_{1}^{2}+x_{1}\left(\;-a_{2}h_{d}+a_{1}\alpha_{3}-{\dot{h}}_{d}\;\right)+a_{1}x_{1}x_{4}. \end{array}$$

Assume

(27) $$\begin{array}{c} \left(\;-a_{2}h_{d}+a_{1}\alpha_{3}-{\dot{h}}_{d}\;\right)=-{k_{1}x}_{1}. \end{array}$$

Equation (26) simplifies to

(28) $$\begin{array}{c} {\dot{f}}_{1}=-a_{2}x_{1}^{2}-k_{1}x_{1}^{2}+a_{1}{x_{1}x}_{4}. \end{array}$$

Equation (27) implies that

(29) $$\begin{array}{c} \alpha_{3}={\left(\;a_{1}\;\right)}^{-1}*\left[\;-k_{1}x_{1}+a_{2}h_{d}+{\dot{h}}_{d}\;\right]. \end{array}$$

**Step 2 .**

To take into account the deviation of the state variable *h*_2_ from the stabilizing function *α*_1_, we define the error variable in (17), taking its derivative,

(30) $$\begin{array}{c} {\dot{x}}_{2}={\dot{h}}_{2}-{\dot{\alpha }}_{1}. \end{array}$$

Substituting (13) in the above equation,

(31) $$\begin{array}{c} {\dot{x}}_{2}=d_{1}h_{2}+d_{2}h_{4}-{\dot{\alpha }}_{1}. \end{array}$$

Putting (17) and (19) in (31),

(32) $$\begin{array}{c} {\dot{x}}_{2}=d_{1}\left(\;x_{2}+\alpha_{1}\;\right)+d_{2}\left(\;x_{4}+\alpha_{3}\;\right)-{\dot{\alpha }}_{1}. \end{array}$$

Again consider the candidate Lyapunov function as

(33) $$\begin{array}{c} {f_{2}=f}_{1}+0.5x_{2}^{2}. \end{array}$$

Its derivative will be

(34) $$\begin{array}{c} {\dot{f}}_{2}={\dot{f}}_{1}+x_{2}{\dot{x}}_{2}. \end{array}$$

Putting (28) and (32) in (34), we get

(35) f˙2=−a2x12−k1x12+d1x22+x2d1α1+d2α3−α˙1+a1x1x4+d2x4x2.

Let

(36) $$\begin{array}{c} d_{1}\alpha_{1}+d_{2}\alpha_{3}-{\dot{\alpha }}_{1}=-k_{2}x_{2}. \end{array}$$

Substituting (36) in (35), we obtain

(37) f˙2=−a2x12−k1x12+d1x22−k2x22+a1x1x4+d2x4x2.

From (36),

(38) $$\begin{array}{c} \alpha_{1}={\left(\;d_{1}\;\right)}^{-1}*\left[\;-k_{2}x_{2}-d_{2}\alpha_{3}+{\dot{\alpha }}_{1}\;\right]. \end{array}$$

**Step 3 .**

As *α*_2_ is the stabilizing function shown in (18), its time derivative will be

(39) $$\begin{array}{c} {\dot{x}}_{3}={\dot{h}}_{3}-{\dot{\alpha }}_{2}. \end{array}$$

Substituting (3) in the above equation,

(40) $$\begin{array}{c} {\dot{x}}_{3}=b_{1}h_{4}-b_{2}h_{3}-{\dot{\alpha }}_{2}. \end{array}$$

Putting (18) and (19) in the above equation,

(41) x˙3=b1x4+α3−b2x3+α2−α˙2,

(42) x˙3=b1x4+b1α3−b2x3−b2α2−α˙2.

Take the candidate Lyapunov function as

(43) $$\begin{array}{c} {\dot{f}}_{3}={\dot{f}}_{2}+x_{3}{\dot{x}}_{3}. \end{array}$$

Putting (37) and (42) in the above equation,

(44) f˙3=−a2x12−k1x12+d1x22−k2x22+a1x1x4+d2x4x2+x3b1x4+b1α3−b2x3−b2α2−α˙2.

After simplification,

(45) f˙3=−a2x12−k1x12+d1x22−k2x22−b2x32+a1x1x4+d2x4x2+x3b1α3−b2α2−α˙2+b1x4x3.

Suppose

(46) $$\begin{array}{c} \left(\;b_{1}\alpha_{3}-b_{2}\alpha_{2}-{\dot{\alpha }}_{2}\;\right)=-k_{3}x_{3}. \end{array}$$

Equation (45) becomes

(47) f˙3=−a2x12−k1x12+d1x22−k2x22−b2x32+a1x1x4+d2x4x2−k3x32+b1x4x3.

From (46),

(48) $$\begin{array}{c} \alpha_{2}={\left(\;b_{2}\;\right)}^{-1}*\left[\;b_{1}\alpha_{3}-{\dot{\alpha }}_{2}+k_{3}x_{3}\;\right]. \end{array}$$

**Step 4 .**

Now taking the error variable shown in (19),

(49) $$\begin{array}{c} x_{4}=h_{4}-\alpha_{3}, \end{array}$$

where *α*_3_ is the stabilizing function. Taking time derivative of the above equation,

(50) $$\begin{array}{c} {\dot{x}}_{4}={\dot{h}}_{4}-{\dot{\alpha }}_{3}. \end{array}$$

Putting (15) in the above equation,

(51) $$\begin{array}{c} {\dot{x}}_{4}=-c_{1}h_{4}+a_{2}h_{1}+b_{2}h_{3}+i\left(\;t\;\right)-{\dot{\alpha }}_{3}. \end{array}$$

Substituting (16), (18), and (19) in the above equation,

(52) x˙4=−c1x4+α3+a2x1+hd+b2x3+α2+it−α˙3,

(53) x˙4=−c1x4−c1α3+a2x1+a2hd+b2x3+b2α2+it−α˙3.

Consider the Lyapunov function as

(54) $$\begin{array}{c} {\dot{f}}_{4}={\dot{f}}_{3}+x_{4}{\dot{x}}_{4}. \end{array}$$

Putting (47) and (53) in the above equation, we get

(55) f˙4=−a2x12−k1x12+d1x22−k2x22−b2x32−k3x32+a1x1x4+d2x4x2+b1x4x3+x4−c1x4−c1α3+a2x1+a2hd+b2x3+b2α2+it−α˙3,f˙4=−a2x12−k1x12+d1x22−k2x22−b2x32−k3x32−c1x42+x4−c1α3+a2x1+a2hd+b2x3+b2α2+it−α3˙+a1x1+d2x2+b1x3.

Let us assume 
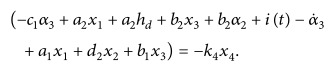
(56)The controlled input for the overall system will be

(57) it=−k4x4+c1α3−a2x1−a2hd−b2x3−b2α2+α˙3−a1x1−d2x2−b1x3.

The derived control law shown in (57) tracks the desired hypnosis level for all the patients undergoing surgery. Simulation results for five patients using this control law which is capable of delivering safe and adequate amount of drug have been shown in the next section. The resulting control law compensates for the patients inherent drug response variability as well as maintaining the hypnosis level within the acceptable range for surgery, as shown in Figure 1. This control law depends on different clinical parameters of the patients undergoing surgery. To investigate and characterize the performance of the designed controller, clinical data including characteristic variables like weight (*W*), height (*H*), and age of five patients is presented in Table 1 [8]. The proposed control law shows improvement in a variety of performance measures with statistical significance.

Based on the patient's attributes, clinical parameters computed using Schneider three-compartmental model for Propofol are given below [25]:

(58) V1=4.27 L,V2=18.9−0.391Age−53 L,V3=238 L,Cl1=1.89+0.0456W−77−0.0681LBM−59+0.0264H−177,Cl2=1.29−0.024Age−53,Cl3=0.836.

Lean body mass (LBM) is a function of patient's gender, height, and weight. For male and female, it is, respectively, given as

(59) LBM=1.1∗W−128∗W2H2,LBM=1.07∗W−148∗W2H2.

The rate constants *q*_10_, *q*_12_, *q*_13_, *q*_21_, *q*_31_ can be calculated as

(60) q10=Cl1V1,q12=Cl2V1,q13=Cl3V1,q21=Cl2V2,q31=Cl3V3.

And Cl_1_, Cl_2_, and Cl_3_ are the clearance of the drug amount from different compartments after metabolism and distribution.

## 4. Results and Discussion

**Figure 3:**
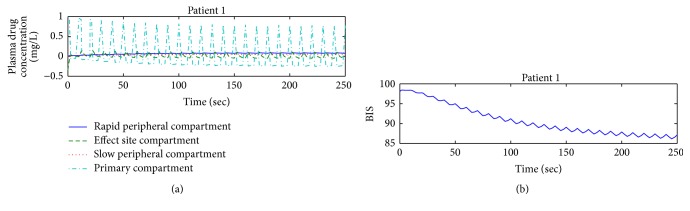
(a) Drug concentration in four compartments and (b) output profile.

This unit presents and discusses the two different conditions of drug infusion for different patients. Controller-less paradigm for patient 1 is shown below demonstrating the plasma-drug concentration in all the four compartments of integrated PKPD model. Simulation results of this controller-less structure display the unusual performance of plasma-drug distribution and elimination in four compartments, as depicted in Figure 3(a), compared to the natural behavior of plasma-drug concentration in all four compartments. The natural behavior of plasma-drug concentration follows the exponential flow (rise and decay of drug amount) in different compartments of PKPD model.  Figure 3(b) indicates the hypnosis level of patient 1 in terms of BIS value which is still far away from the range acceptable for surgery as specified in Figure 1.

Without a controller, Propofol infusion for attaining desired DOH level for surgery can be dangerous and have severe effects on the patient's health and safety during surgery. The result of controller-less model stresses the requirement of a dedicated controller for Propofol administration during and after surgery. With such a classification, the infusion and maintenance of anesthesia entirely rely on anesthesiologist skill and expertise.

The consequences from the above mentioned scheme are not suitable for surgery due to patient's health and safety, as depicted in Figure 3. Employing a controller designed through a nonlinear control strategy, that is, backstepping, brightens the fallouts of the anesthesia system by tracking the desired hypnosis level suitable for surgery.

**Figure 4:**
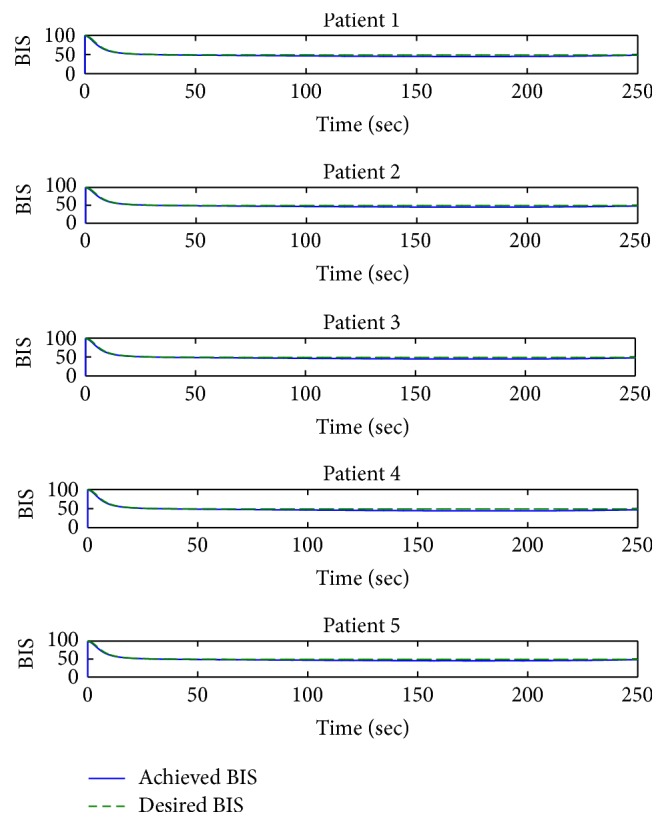
Hypnosis level tracking with backstepping control for 5 patients.

Simulation setup of actual and desired BIS waveforms for the five patients included in Table 1 is shown in Figure 4. The results in above figure show that the hypnosis level of all the five patients depends on different clinical parameters, for example, weight, age, height, gender, and lean body mass. The designed controller handles the interpatient variability together with achieving the desired hypnosis level between 40 and 60 which is suitable for general surgical procedures. BIS waveforms shown in Figure 4 illuminate the robust behavior of the designed controller.

**Figure 5:**
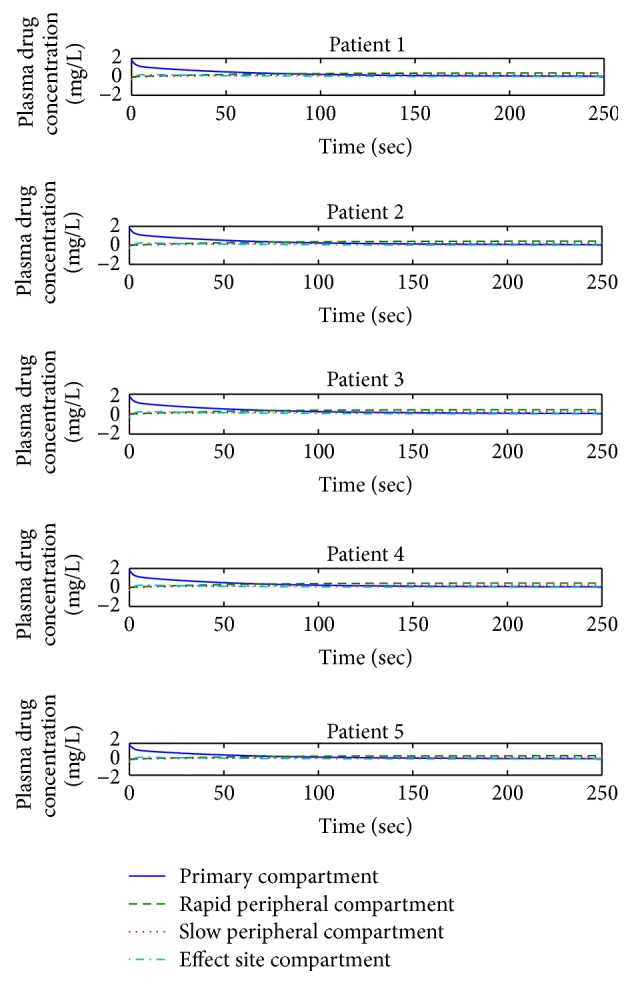
Plasma-drug concentration for 5 patients.

After the drug infusion, the rate of change of plasma-drug concentration with respect to time in all 4 compartments of the integrated PKPD model for 5 patients is shown in Figure 5. As the drug infusion and elimination are done through the primary compartment by using rate constants, the amount of drug is maximum in this compartment initially. After some time the distribution/elimination of drug starts from primary to rapid and slow peripheral compartments in an exponential fashion and the corresponding impact is initiated at the effect site. When the effect site is getting the desired drug concentration, the plasma-drug levels in primary compartment gradually approach to zero.

As the distribution/elimination of the drug from primary compartment to other compartments is carried out in an exponential way, the concentration in the primary compartment decays gradually with time. Similarly, at the same time the plasma-drug concentration in rapid and slow peripheral compartments rises slowly with time. So there exists some time delay from the infusion of the drug to reaching the brain (effect site compartment) which cannot be avoided.

Comparison of different patients on the basis of diverse parameters like age and weight has been shown for analysis. Comparing the drug concentration of patients 4 and 1 illustrated in Figure 5, it is evident that patient 4 being younger exhibits fast metabolism of the drug occurring in primary compartment compared to patient 1. Evaluation of young and old patients reveals that the concentration in rapid peripheral compartment increases substantially due to the fast flow of Propofol from primary compartment. The same effect is replicated in slow peripheral compartment and effect site compartment. The lesser the age of the patient, the faster the metabolism of the drug.

As compared to age, the weight of a patient does not significantly affect the plasma-drug concentration profile. To examine this effect, the drug concentration in patients 2 and 5 (Δweight = 25 kg) has been compared. It has been observed that the concentration of Propofol in the primary compartment of patient 2 decays at reasonably same rate as that of patient 5. Same approach is observed regarding the flow of drug to other compartments of the PKPD model.

**Figure 6:**
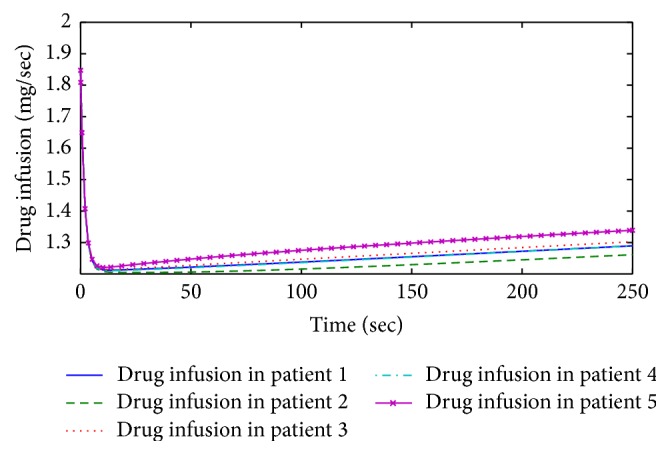
Drug infusion profile for 5 patients.

Figure 6 shows drug infusion profile corresponding to 5 patients which depends on different patient parameters like age, weight, height, gender, and LBM to maintain the desired hypnosis level suitable for surgery. During induction period of the drug, the control law permits the injection of large amount of drug to bring the patient in unconscious state. As the desired depth of hypnosis is attained, the controller retains the specific infusion rate throughout the maintenance phase of anesthesia for each patient for smooth conduction of surgery.

To investigate the effect of patient's weight on drug infusion profile, comparison of patients 2 and 5 is also shown. It is observed that more drug infusion is required for patient 5 (weight = 75 kg) than patient 2 (weight = 50 kg). Same effect is observed for patients 2 and 3.

Form Figures 5 and 6, it is evident that initially in induction phase the drug infusion occurs in large quantity as shown by plasma-drug concentration in primary compartment. After about 5 seconds, the plasma-drug concentration starts decreasing in primary compartment and the drug moves in rapid and slow peripheral and effect site compartment (brain) to make the patient unconscious. The quantity of drug in rapid peripheral compartment blocks the muscle movements of the patient and the part of the drug which moves to effect site compartment makes the patient unconscious. Once the required hypnosis level is achieved for surgery, the specific drug infusion amount is maintained, which in turn maintains the plasma-drug concentration at the effect site during the whole surgical procedure.

**Figure 7:**
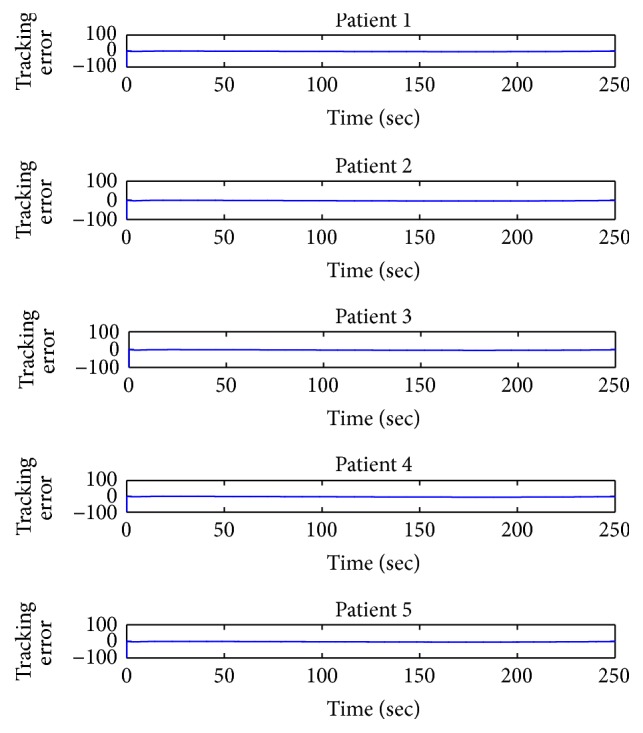
Tracking error for 5 patients in achieving the desired BIS value.

The derived controller tracks the desired value of BIS very well maintaining the tracking error to a very low value confirming the efficacy and effectiveness of the design. Initially, the error in tracking the desired BIS value is maximum, but, after approximately 150 seconds, the controller reduces the tracking error to negligible value proving its accuracy as shown in Figure 7. This scheme shows that the tracking error for all the five patients having different clinical parameters is very small and thus suitable for surgery. Simulation results elucidate the performance of the designed control law with accurate hypnosis level regulation for all the five patients.

## 5. Conclusion

A nonlinear control strategy to explore different aspects of anesthesia infusion scheme with original parameters of five different patients has been proposed in this research study. The designed system without control law shows the uncertain behavior of drug infusion in all four compartments of integrated PKPD mode, approving the requirement of robust controller for Propofol infusion. Backstepping, a nonlinear method, is well known for its robust nature against parameter variations and model uncertainties. The designed control rule tracks the desired conscious level of all five patients in the band suitable for surgery. The stability of the aforementioned control law is examined by means of the Lyapunov theory. This work also shows that the tracking error of the desired and actual BIS values decreases significantly to a very small value when the designed control law is used with the derived patient model shown in Section 2 proving its accuracy and precision. Simulation setup validates the performance of the designed control law with accurate hypnosis regulation for all the five patients shown in Table 1.

With the help of medical professionals at National Institute of Health (NIH) Pakistan, we are going to test the proposed system in real surgical scenario after meeting the medical safety standards. It is imperative to demonstrate practical benefits of this system to convince clinicians.

## Figures and Tables

**Table 1:** Patient's clinical parameters.

Patients	Weight (kg)	Height (cm)	Age	CE_0_	CE_max_	*γ*	*C*_50_
Patient 1	54	163	40	98.80	94.10	2.24	6.33
Patient 2	50	163	36	98.60	86.00	4.29	6.76
Patient 3	58	172	34	96.20	90.80	1.84	4.95
Patient 4	60	164	28	94.70	85.30	2.46	4.93
Patient 5	75	187	37	92.00	104.0	2.10	8.02

## Nomenclature

*h*_1_: Drug amount in rapid peripheral compartment (mg)
*h*_2_: Flow of hypnotic drug at effect site (mg)
*h*_3_: Drug amount in slow peripheral compartment (mg)
*h*_4_: Drug amount in primary compartment (mg)
*q*_10_: Elimination rate constant (sec^−1^)
*q*_1*e*_: Effect site rate constant (sec^−1^)
*q*_12_, *q*_21_, *q*_13_, *q*_31_: Rate constants b/w compartments (sec^−1^)
*q*_*e*0_: Elimination rate constant at effect site (sec^−1^)
*i*(*t*): Infusion rate (mg·sec^−1^)
*γ*: Nonlinear sigmoid curve slope parameter (—)
*C*_*e*_: Concentration at effect site (mg·L^−1^)
*C*_50_: Drug concentration at half of the maximum effect (mg·L^−1^)
CE_0_: Effect in the absence of drug (—)
CE_max_: Maximum effect achieved through drug infusion (—).
